# Early Repair of Aortic Wall Structural Defect by “Net” Endoprosthesis to Arrest the Aneurysm without Interference with Aortic Branch Vessel Perfusion

**DOI:** 10.1055/s-0042-1748842

**Published:** 2022-11-01

**Authors:** Stefano Nazari

**Affiliations:** 1 Department of Research, Fondazione Alexis Carrel, Basiglio (MI), Italy

**Keywords:** aneurysm, dissection, paraplegia, PEARS, TEVAR, prophylaxis, cure

## Abstract

Current treatments of aortic aneurysm include surgical or endovascular, respectively, anatomical or functional, substitution of the aneurysm tract; however, with these methods, perfusion of at least some collateral branches cannot be fully restored, leading to the risk of paraplegia. We present a novel endovascular “net” prosthesis to strengthen the aortic wall while preserving perfusion of collateral branches. This consists of a polyester mesh “net”-layered conduit in a variable cylindrical shape, which is personalized based on patient computed tomography scan images, and is defined by circular crossing spirals of a thin nitinol wire. The prosthetic conduit, shrunk by compressing the nitinol spirals, can be inserted into the vascular lumen and expanded in situ. Then, the insertion control device can be fully removed. Thus, the, “net” prosthesis, positioned inside the aorta in stable contact with the intimal wall for 2 to 5 months, is colonized by neointima and spontaneously moved deeper into the aortic wall in contact with the media, thus being ideally able to stabilize aortic diameter without interference with collateral branch blood perfusion. This new, (ideally) paraplegia-free procedure is aimed at curing the aortic wall structural defect, thus arresting the aneurysm from further progression. This contrasts with current treatments, indicated by aneurysm dimensions for their implied complication risk, which are actually for prophylaxis of impending rupture or dissection rather than fortification of the natural aorta. Moreover, this new approach can be used alongside open surgical procedures (personalized external aortic root support) as well as a frozen “net” elephant trunk technique, for full aortic stabilization.

## Introduction

**Fig. 1:**
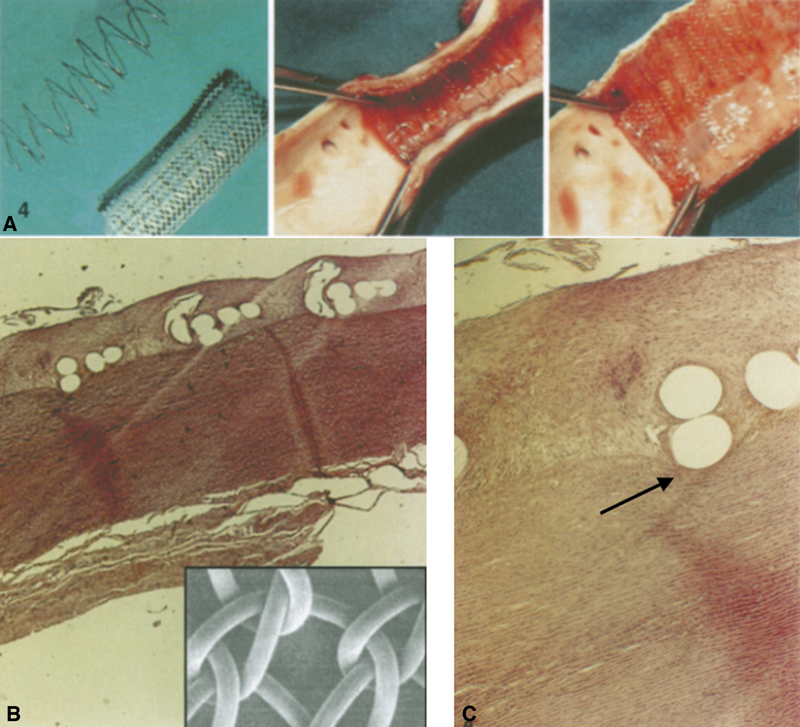
Past experiences 1 showed that polypropylene “Net” had been embodied deep into the vascular wall down to reach the media and appears to be on the predictably physiopathological path of any foreign material away from the blood stream. The net threads of the part not encased up to the media are anyway entirely wrapped by neointima. ( **A** ) The net prosthesis. ( **B** ) The wired net sectioned with the vascular wall exposing the inner vascular lumen. ( **C** ) The vascular wall after removal of the nitinol wires. Blue outlined squares histology of the ( **C** ) blue selection.

The concept of novel endovascular “net” prosthesis may provide safe and early treatment of the root pathology of aneurysms instead of the current approach of prophylaxis or cure of aneurysmal complications. Since 1994, we have reported 1 experimental results of an endovascular “net” prosthesis to strengthen the aortic wall without interference with collateral branch perfusion. Branch vessel is the main source of technical problems and complications of current endovascular procedures. During these nearly three decades, we conceived and mechanically tested this device, both ex vivo 1 and in vivo, in a swine aortic model 2 ( Fig. 1 ). We explored a variety of “net” prosthesis configurations to find those most effective and reliable from a strictly mechanical point of view. We also assessed the most appropriate mesh dimensioning and configuration of the polyester layers, as well as miniaturization of the delivery apparatus to render it suitable for use in the human patient.

The purpose of this article is to illustrate the principles and implications of this new approach, to present our latest endovascular “net” prosthesis devices, and to focus on details learned along this experimental path.

## Background

Looking at the aortic surgery evolution timeline over the past century, some facts in the technological domain of the surgical art could perhaps appear as curious and eventually meaningful.

One of the first attempts at the treatment of an abdominal aortic aneurysm by *cellophane external partial wrapping* was performed (1948) on the most famous theoretical physicist of all time (Einstein). The Carrel vascular suture technique had been developed 46 years earlier (1902). 3 The *first appropriate* successful application of personalized mechanical support of the aorta (precision therapy **P** ersonalized **E** xternal **A** ortic **R** oot **S** upport [PEARS]) was done in 2004. 4

On the contrary, *anatomical or functional full substitution* of the aortic aneurysm tract by a polyester (and/or later on by polytetrafluoroethylene) graft, first introduced in 1951 by DeBakey, is still currently performed, by open or endovascular means, the latter being first applied by Volodos 5 in 1984 and popularized in the western countries by Parodi et al. 6 It must be acknowledged, however, that this current clinical path had been first performed, even in its hybrid setting, in 1982 by H.G. Borst, by his conventional elephant trunk technique. 7

Both open and endovascular full substitution techniques (except the Borst *Conventional Elephant Trunk* ), however, are hampered by potential impairment of the collateral branches of the substituted tract, whose perfusion must generally be restored, either surgically or by appropriate further endovascular procedures. Replacement grafts cannot generally preserve perfusion of the all-important spinal segmental arteries, which are often included in the substituted tract. 8 Flow deprivation induces important anatomical changes in the spinal cord arterial perfusion network, which 9 10 may result in intraoperative or later postoperative paraplegia, despite eventual development of collateral circulation.

Whatever may be the possible causative factors (degenerative, genetic, infective, inflammatory, or traumatic) 11 12 and the related pathophysiological events (proteolysis of the structural components of the aortic wall, inflammation, and abnormal biomechanical forces), aortic aneurysm formation is associated with impairment of two critical structural elements of the aortic wall: elastin and collagen. 11 12 Overall, the gradual reduction of the aortic wall strength causes the most evident feature of aortic aneurysm: dilatation, which, in fact, was first suggested in the external aortic support attempted in the case of Einstein. On the contrary, rupture and dissection, the true clinically relevant adverse events of aortic aneurysm, although beginning with weakness of the aortic wall layers, imply a continuity solution at the inner aortic surface.

## New Target

However, from a merely mechanical point of view, these pathogenic contexts could perhaps indicate total substitution of an aortic aneurysm, either surgically or by endovascular procedures, such an approach may represent *overtreatment* . It may rather be preferable to *strengthen the aortic wall* , by addressing the inner 13 rather than the outer aortic surface. 14 This hypothesis is made obviously easier after the widespread diffusion of endovascular techniques.

From the mere structural mechanic point of view, in fact, inner network support, being applied directly at the origin and peak of the systolic stress, 15 allows more accurate configuration and dimensioning for optimal strengthening support. Moreover, our “net,” positioned inside the aorta in *stable contact* with the intimal wall, is both spontaneously colonized and encased by neointima (as usual for the inner surface of any vascular prosthesis). Also, our very first version of the “net” is spontaneously moved deeper into the aortic wall ( Fig. 1 ), perhaps along the natural pathophysiology path of any intraluminal foreign material.

Our original experimental swine model, in fact, showed migration of roughly one-half of the circumference of the “net” tube into contact with the media layer after a few weeks, 2 well beyond the intimal inner surface ( Fig. 1 ). Although we first thought that the complete “net” neointimal wrapping may have required more time for complete deep incorporation, the real explanation could be instead that the maximal diameter of that “net” conduit, virtually inextensible in that first polypropylene configuration, was simply too small to fully adhere and then to be moved deeper *all around* the inner aortic surface.

This reasoning prompts a very important consideration: contrary to external support devices, 4 14 16 the inner aortic diameter stabilization of our “net” devices does not rely on the maximal diameter of the endovascular “net” prosthesis conduit. Rather, the *mechanical strengthening unit* of our device, in fact, is each and every single mesh whose threads, colonized by fibroblasts, collagen, and other cells, circumscribe and then stabilize the small inner aortic wall area, which also connects to the six confining meshes. Overall, the aortic strengthened area is actually independent from the diameter of the “net” conduit, being stabilized by the threads and the accompanying cellular proliferation process.

This mechanism of aortic stabilization offers a very great advantage compared with the external wrapping of the aorta, allowing a generous oversizing of the maximal geometric diameter of the “net” conduit so that it may achieve the contact with more dilated aortic areas, without accompanying danger or detriment.

## Device Prototypes

**Fig. 2:**
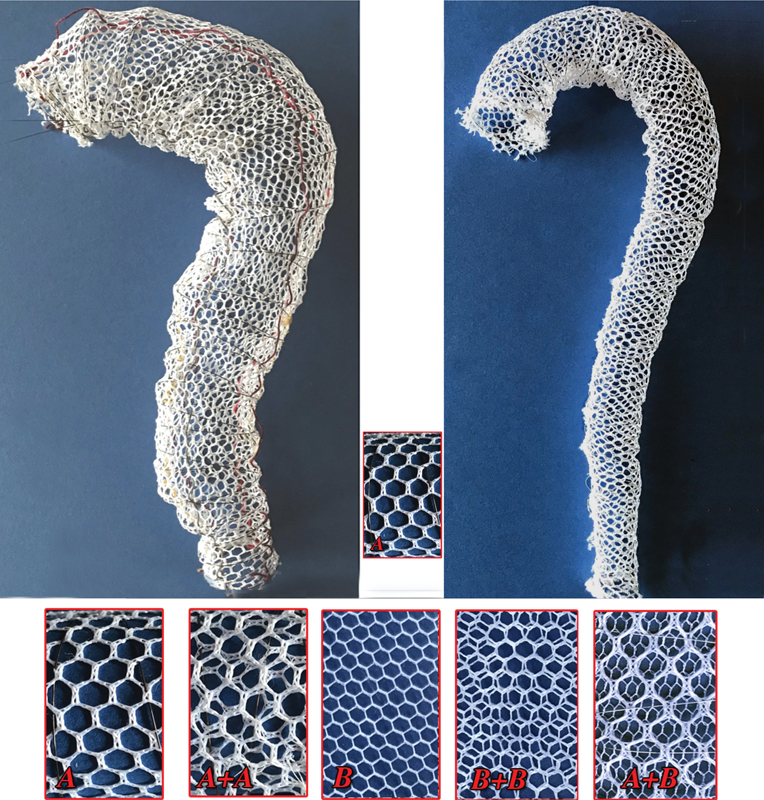
Bottom: The 2 polyester layers ( **A** and **B** ) possible combinations for the prototypes in this experimental phase. From the strict structural mechanics point of view a single polyester layer either with 5 ( **A** ) or 2.5 ( **B** ) mm meshes embodied into the aortic wall with the nitinol wireframe could largely withstand any intravascular stress that may occur. 2 Top: These two prototypes, in the mono-A mesh layers configuration (mandatorily always personalized on the patient computed tomography [CT] scan images), outline the extremes that face this new approach identifying respectively the starting point (left), i.e., giant aortic deformation, and the final goal of this research project (right), i.e., the full prevention of genetic aneurysm and very, very early stabilization of any other aortic aneurysms. Accurate personalization of the device allows the use of a single layer. Contrariwise, current endovascular prosthesis the “net” does not recanalize the blood stream then no/minimal stress at the ends; it is the geometrical shape itself that prevents significant “net” dislocation.

**Fig. 3:**
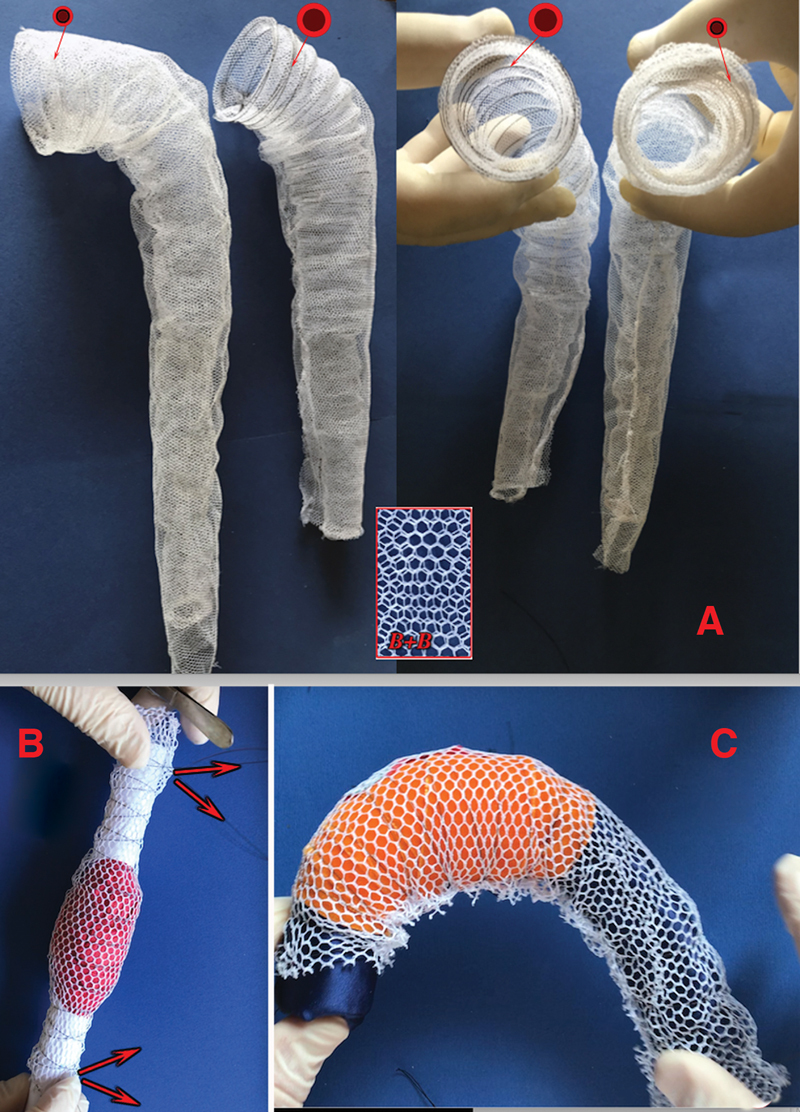
( **A** ) Prostheses with double thinner mesh (B + B) layers as well as stronger (central, shorter) or thinner (external, longer) nitinol wire (smaller or larger red-black circles) that may allow to better comply with inner aortic wall pathology, being perhaps the stronger nitinol wire more appropriate in presence of atherosclerotic pathology and the lighter perhaps more suitable in Marfan and/or in dissection cases. ( **B** , **C** ) The process of personalization of both components of the expandable “net” device is quite simple on the simulation of three-dimensional (3D) printed from patient computed tomography (CT) scan images by pulling out the single nitinol wire at its both ends ( **B** , red arrows).

**Fig. 4:**
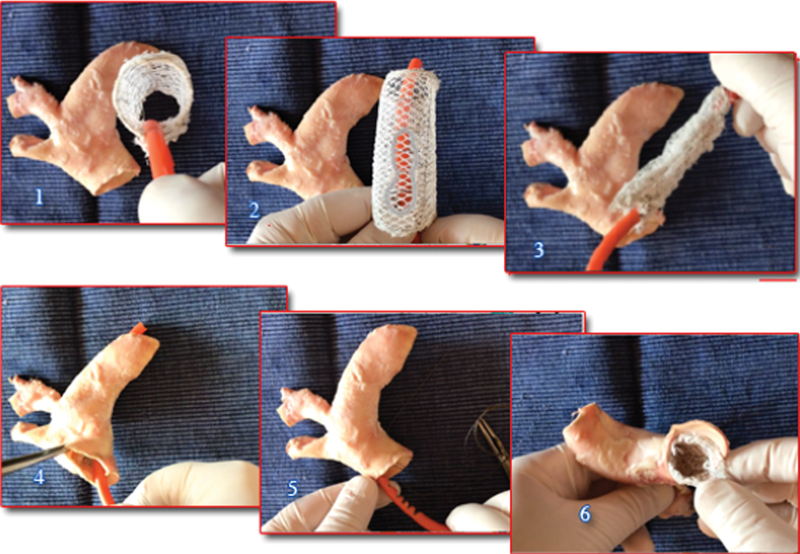
Early prototype of expandable prosthesis for the arch in a bank simulation on the swine model. 1 and 2, the expanded device in double layer; 3, the nitinol wireframe retracted acting simultaneously at each of the 8 nitinol double crossing loops; 4, the device is positioned into the aortic arch; 5, the nitinol wireframe was expanded and the handle of the expanding mechanism withdrawn; 6, appearance of the deployed prosthesis at the distal end. The same mechanical principle for retraction and expansion can be applied, with minor modifications required for control of the selective relevant different amplitude of some loops, for the endovascular positioning of all the nitinol/“net” conduits illustrated.

The latest device consists essentially of three main parts: the polyester network layer(s) ( Fig. 2 , bottom), the support wireframe ( Figs. 2 , top and 3A ) (consisting of a single thin nitinol wire defining a double-crossing spiral loop personalized ( Fig. 3B, C ) at each “net” prosthesis segment, throughout the conduit), and the apparatus for insertion, deployment, and in situ release of the “net” prosthesis ( Fig. 4 ). Device configurations and possible constructive materials may vary according to the vascular location, the local anatomy of the vascular tract, and, perhaps, some other details that further experience would eventually suggest.

The single or double large mesh (≥ 5 mm) layer ( Fig. 2 , lower strip: A, A + A) does not significantly impact the blood flow rate of any aortic collaterals and can be expected to be fully embodied into the aortic wall after having been in contact for 2 to 5 months. Together with the nitinol wireframe, these elements constitute a structural support to the vascular wall that can withstand forces largely exceeding those applied by the bloodstream under clinical circumstances. 2

On the contrary, devices with multiple layer meshes (≤ 1 mm) together with other layers with meshes of various diameters form conduits that could eventually become fully blood-sealed over time. The rate of blood sealing may be pharmacologically controllable before full embodiment into the aortic wall.

It must be emphasized that the primary aim of our approach is *to integrate into the aortic wall* a nitinol-polyester network that restores (or preserves) aortic physiological strength, whose impairment has been (or will be) the cause of the aneurysm. This is very different from providing a new endovascular conduit that recanalizes the blood flow away from the vascular wall, as does any current open or endovascular prosthesis. The “net” prosthesis allows early aortic wall stabilization, ideally without any interference with the perfusion of any collateral branch.

The device configuration may differ in terms of the amplitude of “net” meshes, the number of layers, and the diameter of the nitinol wire whose double-crossing spiral loops define the prosthesis tube shape ( Figs. 2 and 3A ).

It must be emphasized that the nitinol network that defines the tubular shape and keeps the polyester “net” conduit in contact with the intima aortic surface, as well as their respective *different* diameters, *must be personalized based on the three-dimensional model* taken from the patient computed tomography (CT) scan data.

The choice of a single- or double-layer device relies on the accuracy of the CT-based personalization ( Fig. 3B, C ). A double layer would be appropriate only when there is a doubt about the complete adhesion of the “net” to the intima aortic surface throughout the entire aneurysm.

## Comments and Perspectives



**Fig. 5:**
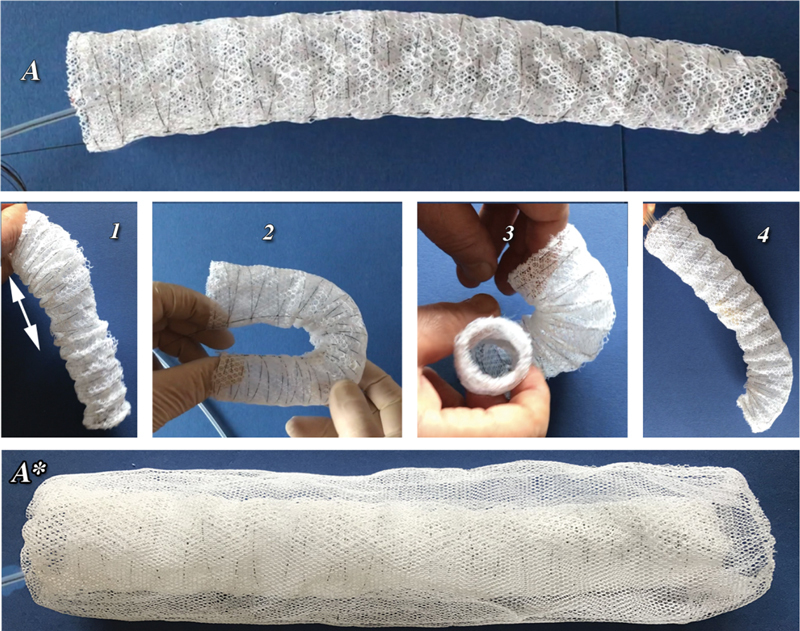
( **A** ) Although been expressly devised as an endovascular procedure this new method may be applied also in virtually any aortic open approach to complete at once the aortic strengthening of the entire extension of the pathology. The prosthesis shown in this very first prototype consists of two layers (A-mesh outside, B-mesh inside) both along connected to the nitinol wireframe. This “net” prosthesis in a hypothesized open thoracic configuration for the descending aorta shows its great adaptability since it preserves the regular lumen amplitude and shape either in variable length (1), in extreme bending (2), as well as in torsion (3); moreover, its tip can be controlled to enhance its progression into the aorta (4). A* : When personalization cannot be performed or whenever it may appear appropriate one or two, quite oversized ( **B** ) (small mashes) layers can be externally added, connected only at the prosthesis ends and free to float in between to enhance contact with the aneurysmal wall.

**Video 1** Device of Fig. 5 .


Our method and device rely on integration into the aortic wall, with the mesh ultimately sequestered from the bloodstream inside the intimal layer and in contact with the media ( Fig. 1 ). The polyester-nitinol framework compensates for the primary aortic wall structural defect, 10 11 without interferences with collateral branch circulation. When clinically proven after refinements in technical details, the endovascular “net” prosthesis will induce radical changes in indications and timing of treatment. In fact, our method corrects the aortic wall structural defect by reestablishing a new structural framework, a mechanical concept already currently in use in other clinical conditions (e.g., hernia repairs).

It must be very clear that the aim of this method is not to offer an alternative therapy to all the aneurysms at the stage where the treatment according to current guidelines is required. On the contrary, in the selected cases (e.g., genetically triggered diseases), the application of this novel therapy may be warranted.

**Fig. 6:**
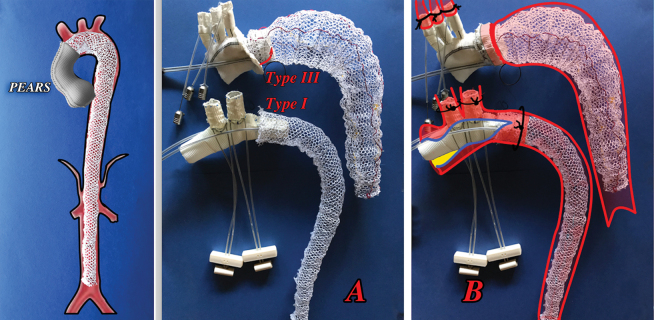
Left: In Marfan and other genetic disorders the full aortic stabilization via transfemoral access can ideally complete PEARS, necessarily open for aortic valve stabilization but without open heart surgery. ( **A** , **B** ) The two “net” prostheses prototypes hypothesized to stabilize descending aorta along the aortic arch open prosthetic substitution. After having positioned the “net” the connection of the proximal end of the descending aorta and with the aortic arch can be done by expandable device type III, 23 (top) or by expandable device 24 25 type I; the latter does not require the full section of the aortic stump and the entire procedure can be completed really very, very quickly (video, https://youtu.be/ZEwzqQevgXw , 16th World Congress WSCTS, Ottawa 2006). The anastomosis with the supra-aortic trunks here is represented as performed with expandable devices type I.

Earlier treatment for endovascular stabilization may be indicated as well when other family members have manifested complications of aortic disease. The “net” treatment could be fully endovascular or part of an open procedure, for example, for aortic root stabilization at the time of aortic valve replacement ( Fig. 5 ). Also, this new endovascular variety of *precision personalized surgery* could be applied as an adjunct to *PEARS* , 4 14 16 17 which is necessarily external to not involve open-heart surgery. The endovascular “net” prosthesis could be positioned up to the arch from femoral access before, during, or after PEARS ( Fig. 6 , left section), thus providing early bimodal aorta stabilization.

This new endovascular method ideally provides the correction of the structural defect 11 12 within the aortic wall with virtually no impact on perfusion of aortic collateral branches.

This can then be viewed as the true therapy of the pathology causing the aneurysm—therapy that can be applied virtually without risk at a very early stage. This would also address those not so rare cases of aneurysms experiencing rupture or dissection before their diameter meets the current criteria for aortic substitution. 18 19 20 21

Endovascular devices implementing this structural support principle, tailored to the vascular tract shape and dynamics, can be hypothesized anywhere in the aorta and, perhaps, eventually also in any other arterial districts.

The essential requisite for the “net” embodiment into the vascular wall is its *stable contact* with the intimal wall for a period of ⅖ months. This requires accurate configuration of the “net” prosthesis *personalized* on patient's CT images; specifically, the diameter of the nitinol wire loops must be predicted on the diameter as the corresponding aortic segment; the “net” conduit that should be 15 to 25% larger.

In addition, when appropriate (because of imperfect personalization of the “net” prosthesis or particularly unfavorable anatomical configuration), further compensation may be provided by a second 15 to 25% larger, external, polyester layer without nitinol wireframe support, simply sutured together only at both ends of the conduit ( Figs. 3 and 5A *) and allowed to float freely to enhance contact with any part of the intimal surface of the aneurysm.

The “net” device should be implicitly immune to endoleak issues of conventional stents. Also, any nitinol wire break in the “net” device should be of little or no consequence.

Although it may appear difficult to correctly keep the “net” device in position (left Fig. 2 ), its geometrical configuration itself implicitly prevents its dislocation even independently from its full embodiment into the aortic wall.

Prevention of age-related aortic elongation (which is significantly consistently observed after thoracic endovascular aortic repair 22 ) is implicit in the working principle of the “net” prosthesis on its target, that is, the stabilization of the aortic wall by strengthening its structure at its media layer. The nitinol/polyester network could act in containing the diameter as well as the length of the aorta where embodied.

A possibly relevant point to be settled by clinical experience concerns the potential effects of our meshes crossing the collateral branches origin. However, both mesh configurations, even together ( Fig. 2 , lower section A + A, B + B, A + B), probably would have little impact on blood flow that could produce turbulence and, perhaps, enhance thrombus formation and microembolization. In our system, it would be simple, if there is a requirement of “stretching” the “net” components in the vicinity of an important branch by endovascular dilation of the mesh elements of that site.

Use of the “net” prosthesis devices is not appropriate in cases of bleeding from full-thickness rupture in any aortic tract. However, in aortic dissection, particularly Type B, the “net” approach could be, perhaps, a safer option than current surgery or endovascular procedures, as the “net” technique can fully preserve spinal cord perfusion.

A very important additional feature is that the “net” technique can be combined with virtually any aortic open approach, through the operative field ( Figs. 5 and 6A, B ) or by endovascular means ( Fig. 6 , left), to provide full aortic strengthening to the pathologic zone(s).

One of the first hypothesized examples could be the open arch-ascending aorta prosthetic replacement where the descending aorta can be fully stabilized with devices as shown in Figs. 5 and 6 ( Video 1 ).

“Net” refinements could include markers allowing to verify appropriate “net” integration into the intimal aortic layer and, perhaps, deeper into the media wall during the postoperative course.

Moreover, It is quite possible that our longitudinally compliant “net” construct may lengthen to accommodate the growth of young subjects. Growing swine is an ideal experimental model to quickly verify if that can occur in a predictable, controlled process that could hopefully open new perspectives in the earlier management of genetic aortic aneurysms.
